# Stratified analysis of carotid plaque and intima–media thickness as stroke predictors in type 2 diabetes

**DOI:** 10.3389/fendo.2025.1718199

**Published:** 2025-12-04

**Authors:** Hongmei Fu, Xianwen Wei, Stefan Wirtz

**Affiliations:** 1 The Department of Endocrinology and Gastroenterology, Friedrich-Alexander University Erlangen-Nürnberg (FAU), Erlangen, Germany; 2 Department of Neurology, Pu’er People’s Hospital, Pu’er, Yunnan, China

**Keywords:** type 2 diabetes mellitus, intima-media thickness, carotid plaque, stroke, vascular risk stratification, subgroup analysis

## Abstract

**Background:**

Stroke represents a common macrovascular complication among individuals with type 2 diabetes (T2DM). Carotid intima-media thickness (IMT) and carotid plaque (CP) serve as key indicators of cerebrovascular pathology. However, it is still unclear whether the associations of IMT and CP with stroke vary across different diabetic patient subgroups. We aimed to investigate the associations of IMT and CP with stroke in T2DM patients and further explored the subgroup variations.

**Methods:**

In this cross-sectional study, 2,627 T2DM patients aged 32–80 years were enrolled. Baseline data including age, sex, anthropometrics, blood pressure, lipid profiles, smoking status, and medication use were collected. IMT and CP were measured by ultrasonography. Logistic regression analysis was used to evaluate their associations with stroke. Stratified and interaction analyses were performed to assess subgroup-specific variations.

**Results:**

Among 2,627 enrolled participants, 51.8% reported CP, 17.8% had increased IMT (>=0.9mm), and 7.8% reported history of stroke. Multivariate analysis demonstrated that IMT and CP were differentially associated with risk for stroke. After adjustment for confounding variables, CP showed stronger associations with stroke (OR = 2.97, 95% CI: 2.04-4.33) compared to IMT. Stratified analyses showed that: (1) IMT had stronger associations with stroke in females (OR = 2.33), older patients (≥60 years) and diabetes duration >10 years; (2) CP showed stronger associations with stroke in males (OR = 3.44), younger patients (≤49 years), and patients with diabetes duration <5 years; (3) CP showed stronger association in smokers, while IMT had higher association with stroke in non-smokers.

**Conclusion:**

Both of the two vascular markers IMT and CP showed associations with stroke in T2DM patients; however, there were significant subgroup variations. Individualized risk assessment in diabetic patients may have important clinical implications. All these findings warranted further prospective studies to confirm the subgroup-specific associations and establish causality.

## Background

Type 2 diabetes (T2DM) affects 10.5% of the global population (536.6 million) and is projected to rise to 12.2% (783.2 million) by 2045 (1). Macrovascular and microvascular complications are common in T2DM, with stroke being a highly prevalent macrovascular outcome (2). Globally, stroke is the second leading cause of death and the third leading cause of disability among individuals with T2DM (3).

A number of studies reported that T2DM is associated with increased stroke risk (4–7). This elevated risk is related to several factors including chronic hyperglycemia (8, 9), dyslipidemia (10), hypertension (11), and accelerated atherosclerosis (12). Carotid artery disease, characterized by increased carotid intima-media thickness (IMT) and carotid plaque (CP), is a well-established marker associated with stroke (13–15). IMT is a marker of subclinical atherosclerosis and the American Heart Association and European Atherosclerosis Society recommend an IMT threshold of ≥0.9 mm for defining IMT abnormality. However, the comprehensive relative associations of IMT and CP with stroke in T2DM patients and how these relationships vary across patient subgroups remains incompletely understood.

Previous studies show that IMT and CP are independently associated with stroke, but few have compared their relative strength of association or explored variations across diabetic subgroups by age, sex, diabetes duration, and metabolic factors. The influence of traditional vascular risk factors like dyslipidemia, smoking, and blood pressure control on the relationship of IMT and CP with stroke is also underexplored.

To address these gaps, we conducted a large-scale cross-sectional study analyzing the independent associations of IMT and CP with stroke in T2DM patients. Stratified and interaction analyses were performed to assess whether the association of IMT and CP with stroke differs by age, sex, diabetes duration, lipid levels, and other metabolic factors. In this study here, we aimed to directly compare the strength of the associations between CP/IMT and stroke in patients with T2DM. We further examined whether these relationships varied across patient subgroups defined by sex, age, diabetes duration as well as key metabolic parameters. Finally, we examined whether lipid parameters (including triglycerides, high density lipoprotein cholesterol, low density lipoprotein cholesterol), smoking status, and blood pressure control status modified the associations between IMT/CP and stroke in T2DM. By illuminating these nuanced, patient-specific patterns, our findings aim to enhance stroke risk stratification in diabetes and thus may help to guide the development of tailored prevention strategies.

## Methods

### Study design and subjects

In this cross-sectional study, we recruited 2,627 T2DM patients aged 32–80 years who had undergone routine medical examination during hospitalization. The exclusion criteria were having malignancy, acute myocardial infarction, acute infection, immune disease, alcohol consumption of *≥*140 g/week (men) or *≥*70 g/week (women), viral hepatitis, and use of hepatotoxic medications. T2DM was diagnosed based on the 2022 American Diabetes Association criteria (16).

Ethical approval was obtained from the Institutional Review Committee of Pu’er People’s Hospital. As the study was limited to retrospective analysis of existing patient data, informed consent was waived.

### Data collection and measurements

#### Clinical data collection

Clinical data, including sex, age, body weight, hypertension, hyperlipidemia, diabetes duration, medical treatments, and smoking history, were extracted from the medical records. Body weight and blood pressure (BP) were measured according to the World Health Organization (WHO) standard protocol. BP was measured in the right upper arm of sitting patients with their feet on the floor and the upper arm supported at the heart level; we measured it twice 5 min apart and took the mean. Body mass index (BMI) was calculated as weight (kg)/height (m^2^).

#### Biochemical measurements

After *≥*8 hours of fasting, venous blood samples were drawn and assayed immediately. Glycated hemoglobin (HbA1c) was assayed by the Tosoh Automated Glycohemoglobin Analyzer (HLC-723G11, Japan); Fasting plasma glucose (FPG) was measured using a Roche automated biochemical analyzer (COBAS 8000 C702, Germany); Lipid profiles (total cholesterol, TC; triglycerides, TG; high density lipoprotein cholesterol, HDL-C; low density lipoprotein cholesterol, LDL-C); and C-reactive protein (CRP), were measured by Roche analyzers (Roche Diagnostics, Mannheim, Germany); Serum insulin were measured by chemiluminescence immunoassay (YHLO Biotech Co., Ltd., Shenzhen, China); hepatitis viral antigens/antibodies were assayed by magnetic microparticle chemiluminescence immunoassay (Xiamen Wantai Kerry Biotechnology Co., Ltd., Xiamen, China). HOMA-IR (homeostatic model assessment) of insulin resistance was calculated as FPG (mmol/L) × FINS (µU/mL)/22.5.

#### Ultrasonography

All ultrasound examinations were performed by certified sonographers using high-resolution real-time scanners (Aplio i700, Canon, Tokyo, Japan). Hepatic steatosis was evaluated by qualified radiologists according to standard criteria: increased hepatic parenchymal echogenicity compared with the kidney or spleen (17).

Carotid ultrasonography was performed via Doppler ultrasound (EPIQ 7C, Philips, Netherlands) using a 3–12 MHz linear transducer. Subjects were examined in the supine position with the head turned 45° in the direction contralateral to the side being examined. Carotid intima-media thickness (IMT) was measured on the far wall of the distal common carotid artery (CCA), about 10–20 mm proximal to the carotid bulb, in a longitudinal plane with no plaques present, according to the recommendations of the Mannheim Carotid IMT Consensus (18). At least three IMT measurements were obtained on each side, and the mean values from both the left and right CCA were used for analysis. Carotid plaques(CP) were defined as focal structures protruding into the arterial lumen by at least 0.5 mm, or 50% more than the surrounding IMT, or with a thickness ≥1.5 mm measured from the media-adventitia interface to the intima-lumen interface.

### Definitions

According to the Chinese BMI classification standard, overweight was defined as 24 to 28 kg/m^2^ (19).

According to the American Diabetes Association (ADA) criteria, hypertension was defined as sustained blood pressure *≥*140/90 mmHg and/or using antihypertensive medications (20). Poor glycemic control was defined as HbA1c level ≥ 7.0% (21); poor cholesterol control as LDL-C level ≥ 100 mg/dL; poor TG control as TG level ≥ 150 mg/dL; poor HDL-C control as HDL-C level ≤ 40/50 mg/dL for men/women (22).

### Statistical analyses

**Figure 1:**
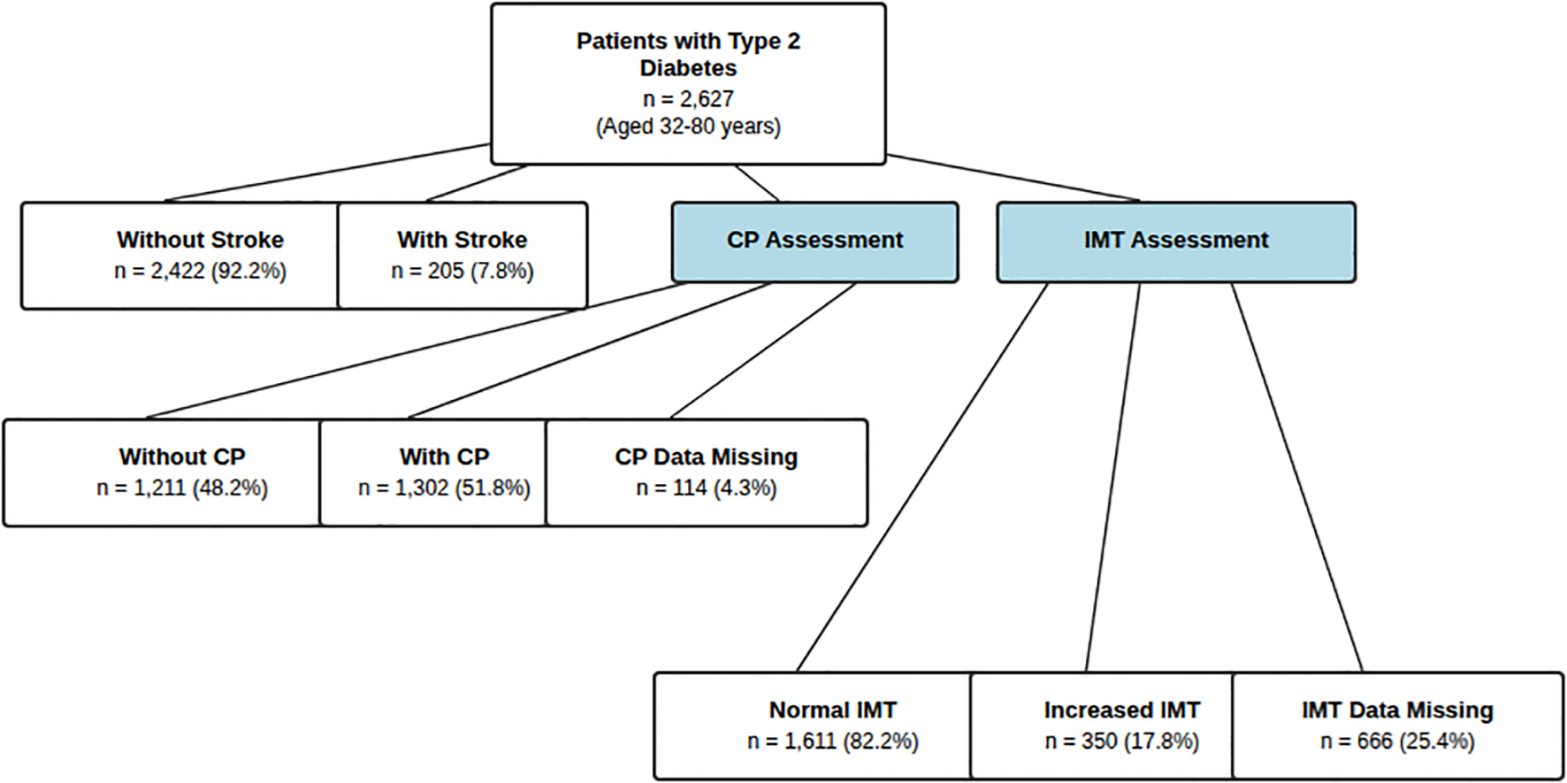
This study included 2,627 patients with type 2 diabetes, employing a two-phase analytical strategy: First phase: Analysis of factors associated with CP and IMT in 2,422 patients without stroke history to minimize reverse causality; Second phase: Analysis of associations between CP/IMT and stroke in all 2,627 patients, examining variations across different subgroups. CP, carotid plaque; IMT, intima-media thickness.

Data analysis was conducted in two phases (**Figure 1**). Phase 1: To examine factors associated with CP and IMT, analyses were limited to patients without prior stroke (n=2,422) to minimize reverse causality bias from treatment modifications after stroke. Of these, 2,319 had valid CP data (1,148 with CP, 1,171 without) and 1,808 had valid IMT measurements (302 with increased IMT, 1,506 with normal IMT) (**Tables 1**, **2**).

**Table 1:** Clinical characteristics of T2DM patients without stroke according to carotid plaque status.

Variable	CP	Without CP	P	P’
	N=1148	N=1171		
Men (%)	710 (61.9)	724 (61.8)	0.992	0.050
Age (year)	58.48 ± 9.46	50.43 ± 10.02	<0.001	<0.001
T2DM duration (year)	8.08 ± 7.24	5.45 ± 6.15	<0.001	0.212
BMI (kg/m^2^)	24.77 ± 3.34	24.95 ± 3.68	0.22	NA
Systolic BP (mmHg)	134.51 ± 20.84	128.66 ± 18.21	<0.001	<0.05
Diastolic BP (mmHg)	81 (73-90)	87 (75-90)	<0.05	<0.05
HbA1c (%)	9.13 ± 2.31	9.42 ± 2.42	<0.05	0.841
HOMA-IR	5.36 ± 13.30	3.93 ± 7.87	0.085	NA
TC (mmol/L)	4.50 ± 1.19	4.60 ± 1.25	0.059	0.487
TG (mmol/L)	2.03 (1.32-3.21)	2.12 (1.35-3.5)	0.071	0.803
HDL-C (mmol/L)	1.05 ± 0.28	1.04 ± 0.28	0.412	0.172
LDL-C (mmol/L)	2.76 ± 0.93	2.73 ± 0.94	0.347	<0.05
CRP	7.19 ± 22.94	6.39 ± 24.82	0.568	NA
IMT (mm)	0.79 ± 0.17	0.73 ± 0.35	<0.001	NA
Smoking (%)	356 (31.0)	313 (26.7)	<0.05	<0.05
NAFLD (%)	522 (45.5)	572 (48.9)	0.106	NA
Anti-diabetic drugs (%)	755 (65.8)	696 (59.4)	<0.05	
Insulin (%)	450 (39.2)	354 (30.2)	<0.001	
Anti-hypertensive drugs (%)	521 (45.4)	319 (27.2)	<0.001	
Lipid-lowering drugs (%)	160 (13.9)	95 (8.1)	<0.001	

Data are expressed as mean ± SD, median (IQR), or counts (percentages) depending on their distribution. “P”values were performed using Student’s t-test or Mann–Whitney test for continuous variables, and χ² test for categorical variables.

“P’ “values represent results from multivariate logistic regression analysis adjusted for sex, Hypoglycemic therapy, Anti-hypertensive drugs, and Lipid lowering drugs.

T2DM, type 2 diabetes mellitus; BMI, body mass index; BP, blood pressure; HbA1c, glycated hemoglobin; HOMA-IR, homeostasis model assessment of insulin resistance; TC, total cholesterol; TG, triglycerides; HDL-C, high density lipoprotein cholesterol; LDL-C, low density lipoprotein cholesterol; CRP, c reactive protein; IMT, intima-media thickness; CP, carotid plaque; NAFLD, non-alcoholic fatty liver disease. NA, not applicable, indicating variables not included in the multivariate regression model due to non-significance in univariate analysis.

**Table 2:** Clinical characteristics of T2DM patients without stroke according to carotid intima-media thickness status.

Variable	Increased IMT(≥ 0.9mm)	IMT(< 0.9mm)	P P’
	N=302	N=1506	
Men (%)	208 (68.9)	908 (60.3)	<0.05 <0.05
Age (year)	58.07 ± 9.71	53.63 ± 10.65	<0.001 <0.001
T2DM duration (year)	8.11 ± 7.31	6.36 ± 6.69	<0.001 0.149
BMI (kg/m^2^)	25.19 ± 3.41	24.82 ± 3.49	0.095 NA
Systolic BP (mmHg)	137.89 ± 21.35	131.16 ± 19.14	<0.001 <0.05
Diastolic BP (mmHg)	83 (74-90)	82 (74-90)	0.205 0.867
HbA1c (%)	9.45 ± 2.28	9.26 ± 2.39	0.213 NA
HOMA-IR	6.79 ± 17.90	3.99 ± 9.55	0.171 NA
TC (mmol/L)	4.67 ± 1.31	4.55 ± 1.17	0.112 0.202
TG (mmol/L)	2.02 (1.35-3.39)	2.11 (1.35-3.44)	0.287 0.062
HDL-C (mmol/L)	1.02 ± 0.25	1.05 ± 0.29	0.072 <0.05
LDL-C (mmol/L)	2.95 ± 1.03	2.74 ± 0.93	<0.001 0.191
CRP	6.54 ± 19.49	5.95 ± 21.66	0.741 NA
CP (%)	220 (72.9)	685 (45.5)	<0.001 NA
Smoking (%)	119 (39.3)	415 (27.6)	<0.001 <0.05
NAFLD (%)	134 (44.4)	726 (48.2)	0.299 NA
Anti-diabetetic drugs (%)	205 (67.9)	934 (62.0)	0.054
Insulin (%)	116 (38.4)	487 (32.3)	<0.05
Anti-hypertensive drugs (%)	123 (40.7)	527 (34.9)	0.058
Lipid-lowering drugs (%)	44 (14.6)	178 (11.8)	0.184

Data are expressed as mean ± SD, median (IQR), or counts (percentages) depending on their distribution.

“P”values were performed using Student’s t-test or Mann–Whitney test for continuous variables, and χ² test for categorical variables.

“P’ “values represent results from multivariate logistic regression analysis adjusted for sex and Hypoglycemic therapy, Anti-hypertensive drugs, and Lipid lowering drugs.

T2DM, type 2 diabetes mellitus; BMI, body mass index; BP, blood pressure; HbA1c, glycated hemoglobin; HOMA-IR, homeostasis model assessment of insulin resistance; TC, total cholesterol; TG, triglycerides; HDL-C, high density lipoprotein cholesterol; LDL-C, low density lipoprotein cholesterol; CRP, c reactive protein; IMT, intima-media thickness; CP, carotid plaque; NAFLD, non-alcoholic fatty liver disease. NA, not applicable, indicating variables not included in the multivariate regression model due to non-significance in univariate analysis.

**Figure 2:**
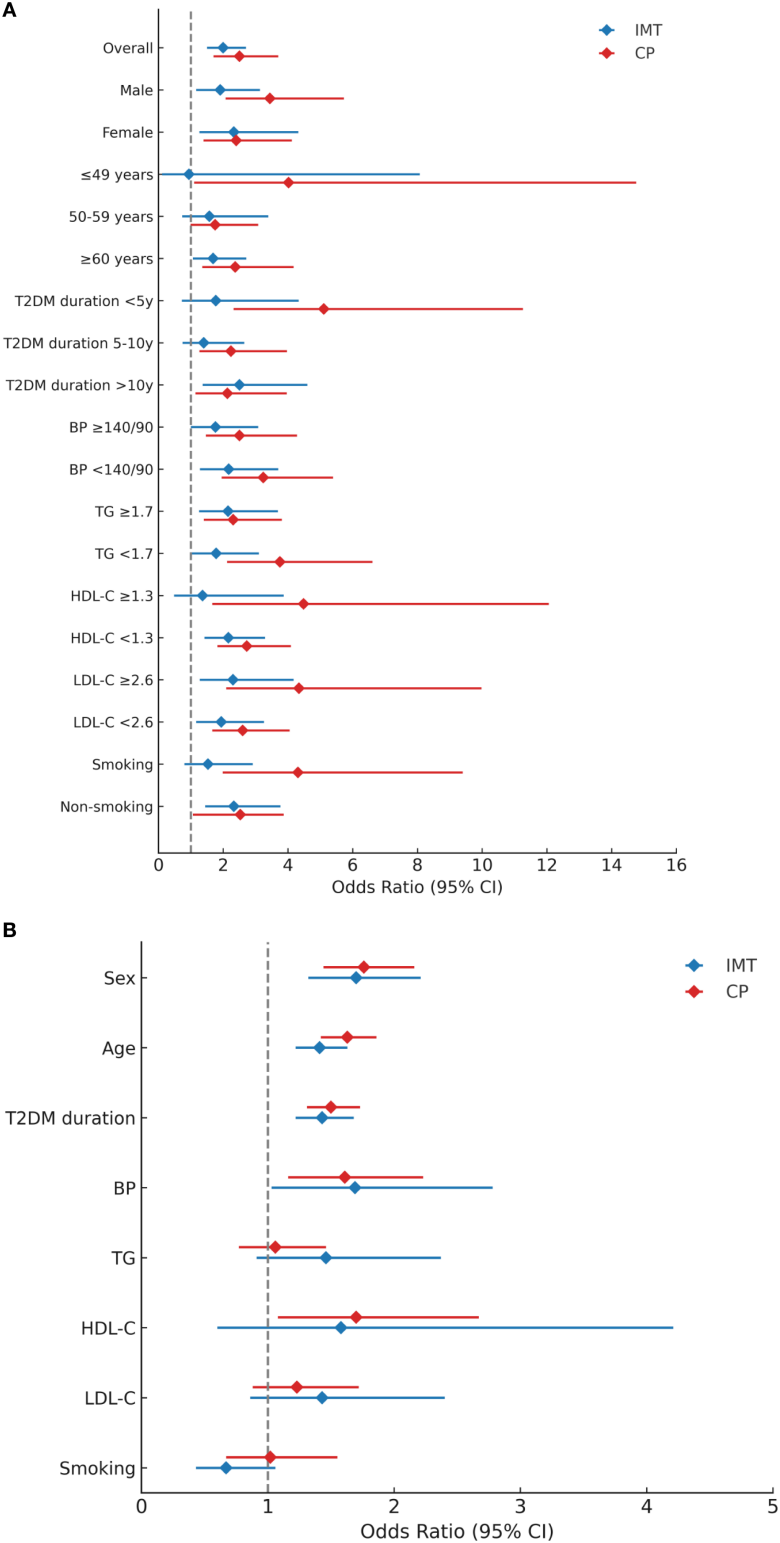
**(A)** Associations of IMT and CP with Stroke Across Subgroups. All models were adjusted for HbA1c, history of hypertension, hypoglycemic therapy, anti-hypertensive drugs, and lipid-lowering drugs. Data are described as odds ratios (95% confidence intervals); CP, carotid plaque; IMT, intima-media thickness. **(B)** Interaction Analysis of IMT and CP with Clinical Factors on Stroke Risk. All data were adjusted for HbA1c, history of hypertension, hypoglycemic therapy, anti-hypertensive drugs, and lipid- lowering drugs. Data are described as odds ratios (95% confidence intervals); CP, carotid plaque; IMT, intima-media thickness; TG, triglycerides; LDL-C, low-density lipoprotein cholesterol.

**Figure 3:**
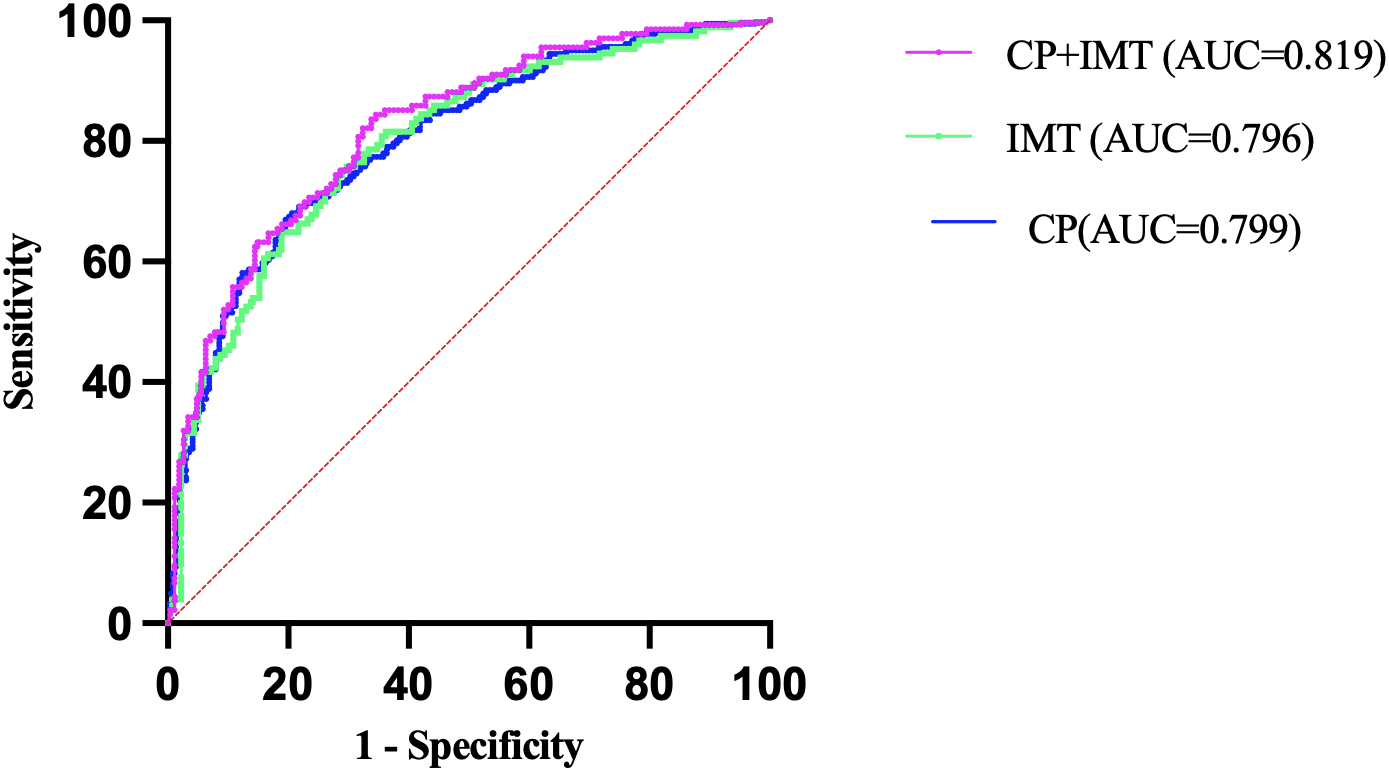
Receiver operating characteristic (ROC) curves comparing the predictive value of carotid intima-media thickness (IMT), carotid plaque (CP), and their combined model for stroke in patients with type 2 diabetes. All models were adjusted for age, sex, SBP, DBP, DM duration, HTN history, HDL-C, LDL-C, smoking,medication use (Hypoglycemic therapy, Anti-hypertensive drugs, and Lipid-lowering drugs).

Phase 2: For the primary objective assessing associations between CP/IMT and stroke all 2,627 patients were included (**Tables 3**–**5**, **Figures 2**, **3**). Valid CP and IMT data were available for 2,513 (missing rate 4.3%) and 1,961 (missing rate 25.4%) patients, respectively.

**Table 3:** Distribution of IMT, CP and stroke prevalence across different patient subgroups.

Variable	N(%)	IMT (mm)	P	CP (%)	P	Stroke (%)	P
Sex			<0.05		0.966		0.128
Male	1617 (61.6)	0.79 ± 0.34		801 (31.9)		116 (4.4)	
Female	1010 (38.4)	0.74 ± 0.15		501 (19.9)		89 (3.4)	
Age (year)			<0.001		<0.001		<0.001
≤49	753 (28.7)	0.71 ± 0.19		191 (7.6)		13 (0.5)	
50-59	989 (37.6)	0.77 ± 0.37		487 (19.4)		63 (2.4)	
≥60	885 (33.7)	0.82 ± 0.22		624 (24.8)		129 (4.9)	
T2DM duration (year)			<0.05		<0.001		<0.001
<5	1190 (45.3)	0.74 ± 0.18		481 (19.1)		44 (1.7)	
5-10	788 (30)	0.79 ± 0.44		413 (16.4)		78 (3.0)	
>10	646 (24.6)	0.79 ± 0.18		407 (16.2)		83 (3.2)	
BMI (kg/m^2^)			0.088		0.622		0.26
≥24	1544 (58.8)	0.78 ± 0.21		757 (30.1))		125 (4.8)	
<24	1030 (39.2)	0.76 + 0.38		515 (20.5)		71 (2.7)	
HbA1c (%)			0.091		0.073		<0.05
≥7	2129 (81)	0.77 ± 0.31		1039 (41.3)		151 (5.7)	
<7	458 (17.4)	0.74 ± 0.15		243 (9.7)		48 (1.8)	
BP (mmHg)			<0.05		<0.001		0.08
≥140/90	1168 (41.2)	0.78 ± 0.35		602 (24.0)		98 (3.7)	
<140/90	1670 (58.8)	0.74 ± 0.20		727 (28.9)		111 (4.2)	
TG (mmol/L)			0.937		0.066		<0.001
≥1.7	1594 (60.7)	0.75 (0.65-0.85)		775 (30.8)		100 (3.8)	
<1.7	1002 (38.1)	0.75 (0.65-0.85)		517 (20.6)		104 (4.0)	
HDL-C (mmol/L)			<0.05		0.374		0.878
≥1.3	426 (16.2)	0.73 ± 0.14		219 (8.7)		34 (1.3)	
<1.3	2164 (82.4)	0.78 ± 0.31		1066 (42.4)		168 (6.4)	
LDL-C (mmol/L)			0.164		0.746		<0.001
≥2.6	1391 (53)	0.78 ± 0.36		688 (27.4)		72 (2.7)	
<2.6	1197 (45.6)	0.76 ± 0.16		598 (23.8)		130 (4.9)	
Smoking (%)			<0.001		<0.05		0.872
Yes	756 (28.8)	0.82 ± 0.46		408 (16.2)		60 (2.3)	
No	1871 (71.2)	0.75 ± 0.15		894 (35.6)		145 (5.5)	

BP, blood pressure; HbA1c, glycated hemoglobin; TC, total cholesterol; TG, triglycerides; HDL-C, high density lipoprotein cholesterol; LDL-C, low density lipoprotein cholesterol.

Data were adjusted for Hypoglycemic therapy, Anti-hypertensive drugs, and Lipid lowering drugs.

P-values have been adjusted for multiple comparisons using Bonferroni correction.

**Table 4:** Stratified analysis of associations between IMT/CP and stroke across different subgroups.

Variable	IMT odds ratio (95%CI)	P	CP odds ratio (95%CI)	P
Sex				
Male	1.91 (1.16-3.13)	<0.05	3.44 (2.07-5.73)	<0.001
Female	2.33 (1.26-4.32)	<0.05	2.40 (1.39-4.12)	<0.05
Age (year)				
≤49	0.94 (0.11-8.07)	0.956	4.02 (1.10-14.76)	<0.05
50-59	1.57 (0.73-3.39)	0.248	1.75 (0.99-3.08)	0.053
≥60	1.69 (1.06-2.71)	<0.05	2.37 (1.35-4.17)	<0.05
T2DM duration (year)				
<5	1.77 (0.72-4.33)	0.212	5.11 (2.32-11.26)	<0.001
5-10	1.40 (0.74-2.65)	0.298	2.24 (1.26-3.97)	<0.05
>10	2.50 (1.36-4.60)	<0.05	2.13 (1.14-3.96)	<0.05
BP (mmHg)				
≥140/90	1.76 (1.00-3.08)	<0.05	2.50 (1.46-4.28)	<0.05
<140/90	2.17 (1.28-3.70)	<0.05	3.24 (1.95-5.39)	<0.001
TG (mmol/L)				
≥1.7	2.15 (1.25-3.69)	<0.05	2.31 (1.40-3.81)	<0.05
<1.7	1.78 (1.02-3.10)	<0.05	3.75 (2.12-6.61)	<0.001
HDL-C (mmol/L)				
≥1.3	1.36 (0.48-3.87)	0.56	4.48 (1.66-12.06)	<0.05
<1.3	2.16 (1.42-3.29)	<0.001	2.73 (1.82-4.09)	<0.001
LDL-C (mmol/L)				
≥2.6	2.30 (1.27-4.17)	<0.05	4.34 (2.09-8.98)	<0.001
<2.6	1.94 (1.16-3.26)	<0.05	2.60 (1.66-4.05)	<0.001
Smoking (%)				
Yes	1.53 (0.80-2.91)	0.20	4.31 (1.98-9.40)	<0.001
No	2.33 (1.44-3.77)	<0.05	2.53 (1.06-3.87)	<0.001

BP, blood pressure; HbA1c, glycated hemoglobin; TC, total cholesterol; TG, triglycerides; HDL-C, high density lipoprotein cholesterol; LDL-C, low density lipoprotein cholesterol.

Data were adjusted for Hypoglycemic therapy, Anti-hypertensive drugs, and Lipid lowering drugs.

**Table 5:** Interaction analysis of demographic and metabolic factors with IMT/CP on stroke risk.

Variable	Odds ratio (95%CI)	Interaction odds ratio (95%CI)	Interaction P
IMT	2.10 (1.43-3.10)		
CP	2.97 (2.04-4.33)		
IMT*Sex		1.70 (1.32-2.21)	<0.001
CP*Sex		1.76 (1.44-2.16)	<0.001
IMT*Age		1.41 (1.22-1.63)	<0.001
CP*Age		1.63 (1.42-1.86)	<0.001
IMT*T2DM duration		1.43 (1.22-1.68)	<0.001
CP*T2DM duration		1.50 (1.31-1.73)	<0.001
IMT*BP		1.69 (1.03-2.78)	<0.05
CP*BP		1.61 (1.16-2.23)	<0.05
IMT*TG		1.46 (0.91-2.37)	0.119
CP*TG		1.06 (0.77-1.46)	0.744
IMT*HDL-C		1.58 (0.60-4.21)	0.356
CP*HDL-C		1.70 (1.08-2.67)	<0.05
IMT*LDL-C		1.43 (0.86-2.40)	0.117
CP*LDL-C		1.23 (0.88-1.72)	0.227
IMT*Smoking		0.67 (0.43-1.06)	0.089
CP*Smoking		1.02 (0.67-1.55)	0.923

IMT, intima-media thickness; CP, carotid plaque; BP, blood pressure; TG, triglycerides; HDL-C, high density lipoprotein cholesterol; LDL-C, low density lipoprotein cholesterol.

Data were adjusted for HBA1c, History of hypertension, Hypoglycemic therapy, Anti-hypertensive drugs, and Lipid lowering drugs.

#### Missing data handling

To evaluate potential bias from missing IMT data, patients with and without IMT measurements were compared on demographic and clinical variables, including stroke prevalence, which was identical (7.8%) in both groups, supporting a “missing at random” assumption (**Supplementary Table 1**). Additionally, missing data were addressed using multiple imputation with the Multivariate Imputation by Chained Equations (MICE) method (**Supplementary Table 2**).

### Statistical methods

Data were analyzed with SPSS software (version 26.0; IBM Corp., Chicago, IL, USA) and GraphPad Prism software (version 9.0; San Diego, CA, USA) and R 4.5. Data normality was assessed with the Shapiro–Wilk test. Normally distributed variables were expressed as means (SD), non-normally distributed variables as medians (IQRs), and categorical variables as frequencies (percentages). Group comparisons were performed using Student’s t-test or Mann–Whitney test for continuous variables, and χ2 test for categorical variables.

Univariate logistic regression identified factors associated with CP/IMT, followed by multivariate models to determine independent predictors. Associations between CP/IMT and stroke were evaluated using ROC curves and AUC values, adjusting for potential confounders (HbA1c, hypertension history, antihypertensive, antidiabetic, and lipid-lowering treatments).

In stratified and interaction analyses, we adjusted for multiple comparisons by applying Bonferroni correction. The p-values and significance levels shown in **Tables 3**, **Table 4** were adjusted by Bonferroni correction. P and significance level values were considered significant when the two-tailed P or P’ <0.05 or <0.001.

## Results

### Baseline characteristics

Among 2,627 T2DM patients (mean age 55.03 ± 10.66 years), 1,617 (61.6%) were men, and the mean diabetes duration was 7.08 *±* 6.97 years. The prevalence of CP was 51.7% (1,302/2,513), increased IMT (*≥*0.9mm) was present in 17.8% (350/1,961) of patients with valid measurements, and 205 (7.8%) had a history of stroke.

### Factors associated with CP and IMT in non-stroke patients carotid plaque

In patients without stroke history (n=2,422), those with CP were significantly older and had longer diabetes duration, higher systolic blood pressure, greater IMT, and higher smoking rates compared to those without CP (all P<0.05). After adjusting for sex and medication use; age, systolic BP, diastolic BP, LDL-C, and smoking remained independently associated with CP (**Table 1**).

### Intima-media thickness

Patients with increased IMT were more likely to be male (68.9% *vs* 60.3%, P<0.05), older (58.07 *±* 9.71 *vs* 53.63 *±* 10.65 years, P<0.001), and had higher systolic blood pressure (137.89 *±* 21.35 *vs* 131.16 *±* 19.14 mmHg, P<0.001) and LDL-C (2.95 ± 1.03 *vs* 2.74 ± 0.93 mmol/L, P<0.001). After multivariate adjustment, age, systolic BP, HDL-C, and smoking remained independently associated with increased IMT(**Table 2**).

### Distribution of vascular markers across subgroups

The distribution of IMT, CP, and stroke varied significantly across different patient subgroups (**Table 3**). Male patients had higher IMT than females (0.79 ± 0.34 *vs* 0.74 ± 0.15 mm, P<0.05). IMT, CP prevalence, and stroke incidence all increased with age and diabetes duration (all P<0.001). Poor blood pressure control and smoking were associated with higher IMT and CP prevalence. Notably, stroke incidence was significantly higher in patients with HbA1c ≥7% compared to those with HbA1c <7% (5.7% *vs* 1.8%, P<0.05).

### Stratified analysis

The associations of IMT and CP with stroke varied markedly across subgroups (**Table 4**, **Figure 2A**). IMT was more strongly linked to stroke in females (OR = 2.33), older individuals (≥ 60 years), and those with diabetes duration >10 years. In contrast, CP showed stronger associations in males (OR = 3.44), younger individuals (≤ 49 years), and those with diabetes duration <5 years.

Both markers remained significantly associated with stroke across all BP levels, with stronger associations in patients with well-controlled BP (OR = 2.17 for IMT *vs*. OR = 3.24 for CP). CP showed a stronger link to stroke in patients with lower TG (OR = 3.75 *vs*. 2.31), elevated LDL-C (OR = 4.34 *vs*. 2.60) and HDL-C (OR = 4.48 *vs*. 2.73). IMT showed similar trends for TG (OR = 2.15 *vs*. 1.78) and LDL-C (OR = 2.30 *vs*. 1.94), but a reversed trend for HDL-C (OR = 1.36 *vs*. 2.16). CP was more strongly associated with stroke in smokers (OR = 4.31 *vs* 2.53 in non-smokers), whereas IMT demonstrated a stronger association in non-smokers (OR = 2.33 *vs* 1.53 in smokers).

### Interaction analysis

After multivariate adjustment, both IMT and CP were independently associated with stroke, with CP showing a stronger overall effect (OR = 2.97) than IMT (OR = 2.10) (**Table 5**).

Significant interactions were observed for both markers with sex, age, diabetes duration, and BP (all interaction P<0.05). No statistically significant interactions were found for TG, LDL-C, HDL-C (except for CP*HDL-C: OR = 1.70, P<0.05), or smoking status (all P>0.05). These interaction patterns are illustrated in **Figure 2B**, which visualizes the differential effects of CP and IMT across clinical subgroups.

## Discussion

This cross-sectional study investigated the associations of carotid IMT and CP with stroke in patients with T2DM, with particular focus on how these associations vary across different patient subgroups. Our key findings include: (1) CP showed stronger overall association with stroke than IMT; (2) these associations demonstrated significant heterogeneity across demographic and metabolic subgroups; and (3) clinically relevant interactions between vascular markers and patient characteristics that support personalized risk assessment approaches.

This study focused exclusively on individuals with T2DM to examine vascular heterogeneity within the disease. Although comparisons with non-diabetic populations could better clarify diabetes-specific contributions to vascular remodeling and stroke risk, our design aimed to capture atherosclerotic patterns unique to T2DM. Future studies including both diabetic and non-diabetic cohorts are needed to validate these findings.

### Comparative value of IMT and CP as stroke risk markers

Our findings demonstrate that both IMT and CP are independently associated with stroke, CP outperforms IMT in stroke prediction which aligns with emerging evidence emphasizing plaque assessment over IMT measurement. The Carotid Plaque-RADS system recently introduced by Saba et al. demonstrated that standardized plaque assessment provides superior stroke risk stratification compared to traditional approaches (23), supporting our observation of CP superiority (OR = 2.97 *vs*. 2.10). Similarly, Ihle-Hansen et al. showed that CP score surpasses traditional risk algorithms in predicting stroke and cardiovascular events (24), consistent with our ROC findings. However, our findings suggest that combining CP and IMT provides only a statistically marginal improvement in predictive performance (AUC = 0.819) over CP (AUC = 0.799) or IMT (AUC = 0.796) alone, indicating limited additive value (**Figure 3**). Nonetheless, their complementary nature may still support joint evaluation in individualized risk stratification. To confirm the robustness of these findings, we additionally performed 10-fold cross-validation using an XGBoost model, which yielded similar AUC values and supported the overall stability of the predictive performance.

Beyond statistical associations, these findings likely reflect distinct vascular phenotypes and pathophysiological mechanisms in T2DM. IMT indicates chronic arterial remodeling from sustained hemodynamic and metabolic stress, while CP rather reflects focal lipid accumulation, inflammation, and plaque instability. Mechanistically, endothelial dysfunction, oxidative stress, and innate immune activation have been shown to drive these changes (25). Emerging evidence shows that metabolic dysregulation in diabetes activates overlapping lipid-glucose-inflammatory pathways that accelerate vascular injury (26), highlighting that IMT and CP are not merely structural markers but manifestations of systemic metabolic-inflammatory stress. This framework clearly supports the subgroup-specific associations observed in our study.

### Sex-specific differences in the associations of IMT and CP with stroke

Our study demonstrated sex-specific differences in the associations of carotid markers with stroke. IMT was more strongly associated with stroke in females (OR = 2.33) whereas CP showed a stronger association in males (OR = 3.44), with statistically significant sex interactions for both markers (**Table 5**).

There may be sex differences in vascular remodeling and atherosclerotic progression. Although the mean IMT was higher in males than in females (0.79 ± 0.34 *vs*. 0.74 ± 0.15 mm, P<0.05), the association between IMT and stroke was much stronger in females. This may suggest that women suffer from greater vascular damage before reaching the threshold for stroke.

At the molecular level, estrogen regulates endothelial function through multiple pathways, such as activating endothelial nitric oxide synthase (27), suppressing NADPH oxidase (28), and inhibiting pro-inflammatory cytokines like IL-6 and TNF-*α* (29). These may be responsible for the different responses of the vasculature in males and females.

Morphologically, men show more focal plaques whereas women show more diffuse arterial wall thickening (30). Recently, a meta-analysis confirmed that males have more lipid-rich, vulnerable plaques as compared to females (31). We added to this knowledge by showing that the different patterns of plaque and stenosis translate into different patterns of stroke risk and thus the rationale base for sex-specific screening and risk stratification.

### Age-related differences in the associations of IMT and CP with stroke

Our stratified analysis identified significant age-dependent variations in the associations of carotid markers with stroke. CP was more strongly associated with stroke in younger patients, whereas IMT showed stronger association in older patients. These differences were statistically confirmed by significant interaction terms between age and both CP and IMT (OR = 1.63 *vs*. 1.41, P<0.001).

This may reflect qualitative differences in atherosclerotic processes across the lifespan. Although the prevalence of CP was lower among younger patients (25.4% ≤49 years *vs*. 70.5% ≥60 years), its presence conferred a markedly higher stroke risk, suggesting that CP in younger diabetic patients may be indicative of a more aggressive, rupture-prone plaque phenotype. Supportingly, Biscetti et al. reported that diabetic patients are predisposed to vulnerable plaque formation, associated with elevated inflammatory biomarkers (32). Similarly, Cao et al. found that insulin resistance promotes vulnerable plaque formation and increases stroke risk (33).

Conversely, the relatively higher IMT-stroke association in older patients may be due to the cumulative vascular damage and age-related arterial remodeling. We found that IMT increased significantly with age (0.71 *±* 0.19 mm in ≤49 years *vs*. 0.82 *±* 0.22 mm in *≥*60 years, P<0.001), indicating that absolute IMT increases may carry different pathological significance across age groups. This may be attributed to the accumulation of advanced glycation end products, which promote arterial stiffness by crosslinking extra-cellular matrix proteins (34, 35). Such age-related vascular remodeling may be responsible for the stronger IMT-stroke association in older diabetic patients.

### Impact of diabetes duration on the associations of IMT and CP with stroke

Our analysis showed that associations of IMT and CP with stroke varied by diabetes duration. CP was significantly more associated with stroke in patients with diabetes duration <5 years (OR = 5.11) and IMT in those with diabetes >10 years (OR = 2.50). The significant interaction terms between diabetes duration and both CP and IMT confirmed these results statistically.

These findings may reflect the heterogeneity of diabetic vasculopathy. Although the prevalence of both IMT and CP increased with diabetes duration (**Table 3**), their associations with stroke differed markedly. In early diabetes, hyperglycemia promotes oxidative stress, inflammation, and lipid accumulation, driving the formation of unstable, rupture-prone plaques (36). Areas of disturbed flow further accelerate these initial vascular changes (37), which may explain why CP-stroke association was significant in patients with shorter diabetes duration.

In contrast, chronic hyperglycemia and inflammation associated with longer diabetes duration may induce thickening and remodeling of arterial walls. Nathan et al. have shown that arterial IMT can be slowed down with intensive glycemic control, suggesting that hyperglycemia may play a direct role in inducing arterial structural changes (38). Similarly, glucose-induced arterial stiffness was closely related to carotid remodeling, reinforcing the idea that prolonged hyperglycemia and inflammation may induce arterial wall thickening and remodeling. Kozakova et al. have shown that glucose-dependent arterial stiffness was closely related to carotid remodeling (39). These findings are consistent with Zhao et al., who found that plaque formation predominates in early diabetes and that IMT becomes prominent with disease duration (40). We generalize this to suggest that these related-over-time vascular phenotypes have different stroke risk relationships according to diabetes duration.

While IMT was generally less predictive of stroke than CP, it exhibited stronger associations within particular subgroups such as women, elderly patients, and those with prolonged diabetes. This may imply that IMT reflects gradual arterial remodeling due to sustained metabolic burden, offering complementary insight alongside the acute pathological features represented by CP.

Prior evidence on the predictive value of IMT has been mixed. While large-scale analyses such as the USE-IMT initiative concluded that common carotid IMT added limited value to traditional cardiovascular risk score (41), other cohort studies have shown that both IMT and plaque burden enhance the prediction of cardiovascular and renal outcomes beyond conventional risk factors (42). Our study contributes to this ongoing debate by highlighting the context-dependent utility of IMT and its potential role in phenotype-specific stroke risk assessment, particularly in situations where plaque evaluation is unavailable or inconclusive.

### Impact of BP control on the associations of IMT and CP with stroke

Contrary to conventional expectations, both IMT and CP showed stronger associations with stroke in diabetic patients with well-controlled BP (ORs: 2.17 *vs* 1.76 for IMT; 3.24 *vs* 2.50 for CP) than in those with poor control (**Table 4**). Interaction analysis confirmed significant effect modification by BP status for both markers (**Table 5**), indicating that BP control influences the relationship between carotid pathology and stroke risk.

Several mechanisms may underlie these counterintuitive findings, though the cross-sectional design limits causal inference. First, in well-controlled patients, carotid abnormalities may represent non-hemodynamic-stressed vessels with intrinsic vascular pathology potentially representing more mature atherosclerosis that may not be reversed by BP control (43, 44). We believe that this may be true since it is possible that well-controlled patients have underlying metabolic/genetic background associated with high stroke risk. On the other hand, patients with more severe hypertension certainly received more aggressive therapy with antihypertensive drugs potentially including drugs with additional cardiovascular protective properties (11), which might be responsible for partial masking of true association in poorly controlled group.

The phenomenon may also relate to the concept of optimal BP in diabetic patients with established vascular disease. Emerging evidence suggests that excessive BP reduction in patients with advanced atherosclerosis may impair perfusion to critical vascular territories, paradoxically increasing stroke risk despite nominal control (45).

These findings should be interpreted with caution due to the cross-sectional design and our use of less stringent BP control criteria (<140/90 mmHg rather than <130/80 mmHg). Unmeasured factors such as control duration, medication types, and BP variability may also contribute to the observed paradox.

### Impact of lipid metabolism on the associations of IMT and CP with stroke

Stratified analyses revealed differences across lipid subgroups (**Table 4**), however the majority were not significantly interacting in the interaction testing (**Table 5**) except for the interaction between CP and HDL-C (OR = 1.70,P<0.05) and found that HDL-C was modifying the association between CP and stroke.

From the stratified analysis, we found that the associations between carotid markers and stroke were stronger in patients with low TG and LDL-C levels, however, these findings were not significantly interacting, which may reflect confounding rather than a true effect modification. The reasons for these results may include: 1. Treatment-Indication Bias: Patients with more extensive carotid disease were more likely to receive lipid-lowering therapy (13.9% with CP *vs* 8.1% without), suggesting that intensive treatment was more likely to have been administered to higher-risk patients, potentially creating inverse associations between lipid levels and stroke risk. 2. Limitations of Traditional Lipid Markers: Traditional measures of lipids may not sufficiently characterize atherogenic risk in diabetic populations. Other markers such as small dense LDL, remnant lipoproteins, and the triglyceride-glucose index, may potentially better reflect insulin resistance and predict stroke risk (33, 46).

The significant CP×HDL-C interaction requires cautious interpretation. The stronger CP-stroke association in patients with higher HDL-C levels challenges the conventional view of HDL as uniformly protective. However, the wide confidence interval (OR = 4.48, 95% CI: 1.66-12.06) and limited sample size (n = 426, 16.2%) indicate substantial uncertainty, underscoring the need for validation in larger cohorts. Several mechanisms may underlie this paradox: In diabetes, HDL-C particles may become dysfunctional through glycation or oxidation, diminishing their atheroprotective functions despite normal or elevated HDL-C levels (47). Additionally, emerging evidence suggests HDL may promote plaque destabilization in advanced atherosclerosis by enhancing cholesterol efflux (48).

These findings highlight the need for more refined lipid-related risk assessment in diabetic vasculopathies. Emerging therapies targeting residual lipid-driven inflammation offer promise beyond traditional LDL-C reduction. For example, RNA-based approaches silencing hepatic PCSK9 has been shown efficacy in reducing residual vascular risk (49), representing a novel strategy for managing lipid-driven atherosclerosis in high-risk populations. Future research integrating advanced lipid biomarkers and evaluating responses to targeted therapies may improve stroke risk stratification and support personalized prevention in T2DM.

### Effect of smoking on the associations of IMT and CP on stroke

Analysis showed that CP was more strongly related to stroke in smokers (OR = 4.31) whereas IMT had a stronger association in non-smokers (OR = 2.33). The interaction term between smoking and these markers did not achieve statistical significance (IMT: OR = 0.67, P = 0.089; CP: OR = 1.02, P = 0.923), however, given the contrasting patterns observed in the stratified analysis, there was suggestive evidence of effect modification.

These findings may be due to the impact of smoking on different atherosclerotic processes. Smokers in our study had higher mean IMT (0.82 ± 0.46 *vs*. 0.75 ± 0.15 mm, P<0.001) and CP prevalence (53.9% *vs*. 47.8%, P<0.05). However, the relationship between these markers and stroke differed by smoking status. The stronger association between CP and stroke in smokers may be results from smoking-driven plaque instability with increased oxidative stress, endothelial dysfunction and inflammation (50). These processes lead to the development of lipid-rich, vulnerable plaques that are more likely to rupture and cause stroke.

Conversely, the stronger IMT−stroke association in non-smokers suggests that in the absence of smoking- related inflammation, IMT may serve as a more specific marker of intrinsic vascular pathology. This is supported by McEvoy et al., who found that smoking increases systemic inflammation (e.g., hsCRP, IL-6) and IMT, while inflammatory status (hsCRP *≥*2 mg/Lmg/L) further amplifies the smoking-IMT association (51). These findings imply that smoking-induced inflammation may obscure the specificity of IMT as an independent vascular risk indicator.

### Clinical implications

Our results have several implications for stroke risk assessment and prevention in patients with T2DM. Use of glucose-lowering therapies, including oral agents and insulin, was more common in participants with CP or increased IMT. This likely reflects confounding by disease duration and metabolic severity, as those requiring intensive treatment typically have longer diabetes history or poorer glycemic control—both established contributors to vascular remodeling. First, subgroup differences in markers’ performance support individualized evaluation strategies. CP may provide better risk stratification in young male subjects with early-stage diabetes, whereas IMT may be more informative in older women with long-standing disease. Second, the associations of vascular markers with demographic factors further demonstrate the need for population-specific stroke risk models, and suggests that improvements in predictive accuracy may be achieved by adding age, sex, and years since diagnosis.

Third, the results emphasize the need to move away from the practice of applying a single vascular marker to all patients. The combined model (AUC = 0.819) offered a slight improvement over either CP or IMT alone, supporting a complementary role for both in stroke risk evaluation. Recent developments have enabled the practical implementation of comprehensive carotid evaluation in clinical care, e.g., the use of the standardized Plaque-RADS system (23).

In summary, our results suggest that stroke prevention in T2DM can be guided by patient risk stratification: namely, identification of high-risk subgroups, selection of appropriate vascular markers, and application of targeted interventions.

### Study limitations and future directions

This study has several limitations. First, the small number of stroke cases may have reduced statistical power for subgroup comparisons. However, baseline demographic and metabolic characteristics were generally comparable between stroke and non-stroke participants, minimizing potential bias. In addition, its cross-sectional design precludes causal inference. Although we applied a two-phase analytical strategy to reduce reverse causality, it remains possible that stroke led to treatment modifications (e.g., intensified antihypertensive or lipid-lowering therapy), which in turn affected CP progression and IMT values thereby attenuating or confounding observed associations.

Second, CP assessment followed a binary scheme (presence/absence) without detailed evaluation of plaque morphology, echogenicity, or vulnerability. Prior studies have shown that these plaque characteristics independently predict stroke risk beyond presence alone (52–54), which may partially explain some of the heterogeneity in our findings.

Third, residual confounding cannot be completely ruled out by multivariate adjustment for medication type, dose, and compliance. The much greater prevalence of lipid-lowering therapy in CP patients (13.9% *vs*. 8.1%) indicates the possible presence of treatment bias influencing lipid associations.

Fourth, we applied a less strict BP control threshold (<140/90 mmHg *vs*. <130/80 mmHg) which might have affected our interpretations of BP-related analyses. Furthermore, the high rate of missing IMT data (25.4%) may have introduced bias despite the missing-at-random assumption and multiple imputation. Inflammatory biomarkers (CRP, IL-6, TNF-α) were not routinely measured, limiting analysis of inflammation-related mechanisms. Among stroke-free participants, CRP showed no significant association with carotid markers, and its high degree of missingness further reduced analytical value; these markers were therefore excluded from multivariable models. Future studies incorporating high-sensitivity CRP and cytokine panels may help to better identify the inflammatory pathways connecting metabolic stress to vascular remodeling in T2DM. Finally, our study subjects were hospitalized patients, who probably represent more advanced or less well-controlled T2DM patients, and thus, our results may not be generalizable to an outpatient or early-stage population.

Future research should employ prospective designs in investigating IMT/CP progression and their associations with stroke as well as add plaque morphology and vascular function markers to improve risk prediction. With respect to metabolic–vascular interaction, further studies are warranted to investigate insulin resistance and vascular injury. Personalized interventions according to patients’ profiles could be an interesting field for future research for which the emerging field of artificial intelligence offers valuable applications for CP analysis and stroke prediction.

Although glycemic indicators (HbA1c) were adjusted in the multivariate models, further stratification by metabolic control could provide additional insight into vascular remodeling dynamics. Chronic hyperglycemia and insulin resistance may amplify the impact of IMT and plaque on cerebrovascular risk through endothelial dysfunction and low-grade inflammation. Future studies integrating metabolic stratification are warranted to clarify these mechanisms.

## Conclusion

This cross-sectional study demonstrates that both IMT and CP are independently associated with stroke in T2DM patients, with notable subgroup differences: CP showed stronger associations in males, younger patients, and early-stage diabetes, whereas IMT was more predictive in females, older individuals, and those with long-standing disease.

These findings challenge uniform screening strategies and support phenotype-driven risk stratification, highlighting the need for prospective validation to inform targeted prevention. Future prospective studies should further refine IMT cut-off values by gender and age to improve individualized vascular risk assessment in diabetes.

## Data Availability

The raw data supporting the conclusions of this article will be made available by the authors upon reasonable request.
