# Supplementary material for: Stratified analysis of carotid plaque and intima–media thickness as stroke predictors in type 2 diabetes
Source: Front Endocrinol (Lausanne). 2025 Dec 4;16:1718199. doi: 10.3389/fendo.2025.1718199 (PMC12711504; doi:10.3389/fendo.2025.1718199)
Supplement: Supplementary file 1 [file Table1.docx]

Supplementary Table 1. Comparison of baseline characteristics in patients with and without valid IMT measurements.

| \|  \| IMT \| Without IMT \| P \| \| --- \| --- \| --- \| --- \| \|  \| N=1961 \| N=666 \|  \| \| Men (%) \| 1202(61.3%) \| 415(62.3%) \| 0.641 \| \| Age (year) \| 55.05±10.75 \| 54.96±10.39 \| 0.847 \| \| T2DM duration (year) \| 6.95±6.92 \| 7.46±7.11 \| 0.104 \| \| BMI (kg/m2) \| 24.88±3.43 \| 24.89±3.61 \| 0.961 \| \| Systolic BP (mmHg) \| 132.56±19.84 \| 129.47±19.76 \| <0.05 \| \| Diastolic BP (mmHg) \| 82(74-90) \| 81(74-90) \| 0.792 \| \| HbA1c (%) \| 9.24±2.37 \| 9.20±2.36 \| 0.693 \| \| HOMA-IR \| 4.43±11.05 \| 5.06±9.46 \| 0.448 \| \| TC (mmol/L) \| 4.53±1.21 \| 4.42±1.28 \| <0.05 \| \| TG (mmol/L) \| 2.06(1.33-3.37) \| 1.98(1.29-3.10) \| 0.076 \| \| HDL-C (mmol/L) \| 1.04±0.29 \| 1.04±0.29 \| 0.927 \| \| LDL-C (mmol/L) \| 2.75±0.96 \| 2.62±0.90 \| <0.05 \| \| Stroke(%) \| 153(7.8%) \| 52(7.8%) \| 0.996 \| \| CP (%) \| 1031(52.6%) \| 271(40.7%) \| 0.135 \| \| Smoking (%) \| 586(29.9%) \| 170(25.5) \| <0.05 \| \| NAFLD (%) \| 901(45.9%) \| 287(43.1%) \| 0.793 \| \| Anti-diabetetic drugs (%) \| 1250(63.7%) \| 421(63.2%) \| 0.806 \| \| Insulin (%) \| 682(34.8%) \| 274(41.1%) \| <0.05 \| \| Anti-hypertensive drugs (%) \| 744(37.9%) \| 265(39.8) \| 0.396 \| \| Lipid-lowering drugs(%) \| 292(14.9%) \| 52(7.8%) \| <0.001 \| |
| --- | --- | --- | --- | --- | --- | --- | --- | --- | --- | --- | --- | --- | --- | --- | --- | --- | --- | --- | --- | --- | --- | --- | --- | --- | --- | --- | --- | --- | --- | --- | --- | --- | --- | --- | --- | --- | --- | --- | --- | --- | --- | --- | --- | --- | --- | --- | --- | --- | --- | --- | --- | --- | --- | --- | --- | --- | --- | --- | --- | --- | --- | --- | --- | --- | --- | --- | --- | --- | --- | --- | --- | --- | --- | --- | --- | --- | --- | --- | --- | --- | --- | --- | --- | --- | --- | --- | --- | --- |

Values are presented as mean ± SD or median (IQR) for continuous variables, and number (percentage), as appropriate.

P-values were calculated using Student’s t-test for normally distributed continuous variables, Mann–Whitney U test for skewed variables, and chi-square test for categorical variables.

Supplementary Table 2. Logistic regression analysis of IMT and CP for stroke before and after multiple imputation.

| Variable | OR Before Imputation | 95% CI | P | OR After Imputation | 95% CI | P |  |
| --- | --- | --- | --- | --- | --- | --- | --- |
| IMT | 1.64 | 1.09–2.45 | <0.05 | 1.81 | 1.17–2.78 | 0.007 |  |
| CP | 3.28 | 2.11–5.11 | <0.001 | 2.38 | 1.63–3.48 | <0.001 |  |

Multiple imputation was conducted using MICE (20 iterations). Covariates were selected based on clinical/statistical relevance. Associations remained significant post-imputation.
